# Trends in refractive surgery at an academic center: 2007-2009

**DOI:** 10.1186/1471-2415-11-11

**Published:** 2011-05-14

**Authors:** Irene C Kuo

**Affiliations:** 1 Wilmer Laser Vision Center, Wilmer Eye Institute, Department of Ophthalmology, Johns Hopkins University School of Medicine, Baltimore, MD, USA

## Abstract

**Background:**

The United States officially entered a recession in December 2007, and it officially exited the recession in December 2009, according to the National Bureau of Economic Research. Since the economy may affect not only the volume of excimer laser refractive surgery, but also the clinical characteristics of patients undergoing surgery, our goal was to compare the characteristics of patients completing excimer laser refractive surgery and the types of procedures performed in the summer quarter in 2007 and the same quarter in 2009 at an academic center. A secondary goal was to determine whether the volume of astigmatism- or presbyopia-correcting intraocular lenses (IOLs) has concurrently changed because like laser refractive surgery, these "premium" IOLs involve out-of-pocket costs for patients.

**Methods:**

Retrospective case series. Medical records were reviewed for all patients completing surgery at the Wilmer Laser Vision Center in the summer quarter of 2007 and the summer quarter of 2009. Outcome measures were the proportions of treated refractive errors, the proportion of photorefractive keratectomy (PRK) vs. laser-assisted in-situ keratomileusis (LASIK), and the mean age of patients in each quarter. Chi-square test was used to compare the proportions of treated refractive errors and the proportions of procedures; two-tailed t-test to compare the mean age of patients; and two-tailed z-test to compare proportions of grouped refractive errors in 2007 vs. 2009; alpha = 0.05 for all tests. Refractive errors were grouped by the spherical equivalent of the manifest refraction and were considered "low myopia" for 6 diopters (D) of myopia or less, "high myopia" for more than 6 D, and "hyperopia" for any hyperopia. Billing data were reviewed to obtain the volume of premium IOLs.

**Results:**

Volume of laser refractive procedures decreased by at least 30%. The distribution of proportions of treated refractive errors did not change (p = 0.10). The proportion of high myopes, however, decreased (p = 0.05). The proportions of types of procedure changed, with an increase in the proportion of PRK between 2007 and 2009 (p = 0.02). The mean age of patients did not change [42.4 ± 14.4 (standard deviation) years in 2007 vs. 39.6 ± 14.5 years in 2009; p = 0.4]. Astigmatism-correcting IOL and presbyopia-correcting IOL volumes increased 15-fold and three-fold, respectively, between 2007 and 2009.

**Conclusions:**

Volume of excimer laser refractive surgery decreased by at least 30% between 2007 and 2009. No significant change in mean age or in the distribution of refractive error was seen, although the proportion of high myopes decreased between summer quarters of 2007 and 2009. PRK gained as a proportion of total cases. Premium IOL volume increased, but still comprised a very small proportion of total IOL volume.

## Background

In October 1999, VISX (Abbott Medical Optics, Santa Ana, CA) reported 1 million procedures had been performed in the United States using their excimer platform http://www.visx.com/corporate/company_overview/history.php; today, the company reports 6 million procedures have been performed with their lasers in the US. Intralase (AMO, Santa Ana, CA) became the first femtosecond laser approved by the Food and Drug Administration (FDA) for use in laser-assisted in-situ keratomileusis (LASIK) in 2001. To date, 1 million procedures have been performed using this laser. In 2003, VISX CustomVue, wavefront-guided technology based on Hartmann-Shack aberrometry, gained approval by the FDA. Despite these milestones in technology and volume, the market for refractive surgery has changed since these approvals. One reason may be that the United States officially entered a recession in December 2007, according to the National Bureau of Economic Research (NBER) (http://www.nber.org/cycles/dec2008.pdf). In addition, there was much press coverage in April 2008 when the FDA convened a public advisory panel of outside experts to listen to patients unhappy with the results of their LASIK surgery and to consider how to improve information for patients and physicians about LASIK http://www.fda.gov/MedicalDevices/ProductsandMedicalProcedures/SurgeryandLifeSupport/LASIK/UCM061421.

It is the impression of some surgeons that the clinical characteristics of patients seeking evaluation and undergoing refractive surgery have changed over this time. One possible reason is that early adopters have already undergone surgery, and now surgeons are seeing more patients with characteristics that might have disqualified them from surgery in the past using older technology. It is estimated that more than 60 million people are eligible for excimer laser refractive surgery in the US alone http://www.visx.com/corporate/company_overview/history.php, meaning only 10% of the market has been penetrated. Even if the market is a fraction of the estimate, given the amount of capital investment needed for to perform refractive laser surgery, it is of interest to surgeons (and market-watchers) to know the type of patient undergoing refractive surgery today. The purpose of this study was to determine whether patient characteristics and procedure type have changed between 2007 and 2009 at one academic center. A secondary goal was to determine whether the volume of premium (astigmatism- or presbyopia-correcting) intraocular lenses (IOLs) has changed in the same period, since patients have to pay for these IOLs out-of-pocket as they do for laser refractive surgery.

## Methods

This chart review was conducted under a protocol approved by the Institutional Review Board of Johns Hopkins University and conducted in accordance with the tenets of the Declaration of Helsinki. The Wilmer Laser Vision Correction Center, as part of the Refractive Surgery Service at Wilmer Eye Institute, has maintained a prospective database of all patients who had laser refractive surgery since January 1, 1997. All refractive surgical candidates undergo a thorough preoperative evaluation, including detailed medical, ocular and social history; preoperative uncorrected distance visual acuity (UDVA); best spectacle-corrected distance visual acuity (BSCVA); corneal topography (Zeiss Humphrey Meditec) and Orbscan (Bausch & Lomb); corneal pachymetry by ultrasound and by Orbscan; biomicroscopic examination; pupillary examination; Schimer testing; dilated examination; cycloplegic refraction; and cycloplegic visual acuity. All cases of PRK and LASIK were wavefront-guided using the VISX Star S4 CustomVue platform with iris registration; the eye tracker and iris registration were engaged in treatment of all eyes.

Clinical characteristics of patients completing excimer laser refractive surgery [LASIK, phototherapeutic keratectomy (PTK), or photorefractive keratectomy (PRK)] in the summer quarter (July, August, September) of 2007 and the same quarter in 2009 were de-identified and examined. Age, uncorrected visual acuity, manifest refraction, and surgical plan (intended treatment parameters, whether primary or enhancement procedure, and type of procedure--PRK, LASIK, or PTK) were extracted. Spherical equivalent of the manifest refraction was chosen as the means by which to categorize patients: "low myopia" for 6 diopters of myopia or under, "high myopia" for more than 6 diopters of myopia, and "hyperopia" for any amount of hyperopia.

Billing records were used to track the volume of premium lenses implanted throughout Wilmer because patients (not insurance companies) pay for these lenses.

A three-month time frame was chosen to attain enough patients and to compensate for random fluctuations that could occur within a shorter time frame. The summer quarter of 2007 was chosen because it preceded the official start of the economic recession and because there is an impression that the economic downturn has affected not only the volume of surgery, but also the clinical characteristics of surgical patients.

### Statistical analysis

The chi-square test was performed to compare the over all distribution of treated refractive errors (low myopia, high myopia, hyperopia) in the summer quarter of 2007 with the distribution in the same quarter of 2009. The z-test was used to compare the proportion of each group of refractive error (low myopia, high myopia, hyperopia) in 2007 with the respective proportion in 2009 in the same quarter.

The chi-square test was used to compare the distribution of procedures (PRK or LASIK) in 2007 with that in 2009. It was also used to compare the distribution of PTK and non-PTK procedures in 2007 and 2009. The z-test was used to compare the proportion of procedures that were enhancements in 2007 vs. 2009. The proportion was calculated as the number of enhancement procedures divided by the total number of procedures in that quarter. The t-test was performed to compare the mean age of patients undergoing surgery in 2007 with that of patients having surgery in 2009. Both the z-test and t-test were two-tailed. A p-value of < 0.05 was considered significant for all tests.

## Results

Surgical volume decreased by at least 30%. The distribution of proportions of refractive errors of patients who completed refractive surgery is displayed in Table 1. The change in distribution of proportions between the summer quarters of 2007 and 2009 was not statistically significant (chi-square = 4.53, 2 degrees of freedom, p = 0.10). Examining each group of refractive error between 2007 and 2009, the proportion of high myopes changed (z = 2, p = 0.05), but the proportions of hyperopes and low myopes did not change (z = 1.1, p = 0.3; z = 0.9, p = 0.4, respectively).

**Table 1:** Proportion of refractive errors of patients who underwent excimer laser refractive surgery in the summer quarter of 2007 and the summer quarter of 2009

Refractive error	2007	2009
**Low myope**		

**High myope**		

**Hyperope**	12%	17%

Excimer laser refractive surgery was comprised of photorefractive keratectomy and laser-assisted in-situ keratomileusis. "Low myope" was defined as patients whose spherical equivalent of the manifest refraction was 6 diopters or less of myopia; "high myope" was defined as spherical equivalent of the manifest refraction that was worse than 6 diopters of myopia; hyperope is defined as any amount of hyperopia. (Percentages add up to less than 100% in 2009 because of rounding).

The distribution of proportion of procedures that were PRK or LASIK is shown in Table 2. The distribution of procedures changed between 2007 and 2009 (chi-square = 5.45, 1 degree of freedom, p = 0.02), with an increase in the proportion of PRK. A survey of the laser logs between 2000-2009 showed that the proportion of PRK to total volume of surgery increased from 3% in 2003 to 7% in 2006 to 17% in 2008 to 29% in 2009. PTK comprised 5.6% of all refractive surgery cases in the summer quarter of 2007, and it comprised 1.3% of cases in the summer quarter of 2009, the remainder of cases being non-PTK procedures (PRK and LASIK, primary procedures and enhancements). The distribution of proportions of PTK vs. non-PTK cases changed between 2007 and 2009 (chi-square = 13.35, 1 degree of freedom, p < 0.001).

**Table 2:** Proportion of patients who underwent photorefractive keratectomy (PRK) vs. laser-assisted in-situ keratomileusis (LASIK) in summer quarter of 2007 and in the summer quarter of 2009

Procedure type	2007	2009
**PRK**	17%	29%

**LASIK**	83%	71%

The percentage of enhancements (either PRK or LASIK) increased from 4.8% in 2007 to 13.3% in 2009 (z = 2.40, p = 0.016). In 2007, enhancements were done predominantly in patients who had undergone LASIK previously for high myopia; the remaining 25% previously had low myopia. In 2009, 50% of enhancements were done patients who had undergone LASIK for low myopia, 25% on eyes with previous hyperopia, and 25% on eyes with previous high myopia.

There was no significant difference in the mean age of patients undergoing surgery (LASIK, PRK, or PTK). The mean age in 2007 was 42.4 ± 14.4 (standard deviation) years, and the mean age in 2009 was 39.6 ± 14.5 years (t = 1.02, p = 0.4).

The number of astigmatism-correcting IOLs increased 15-fold, and the number of presbyopia-correcting IOLs increased three-fold between 2007 and 2009. Overall premium IOL volume increased five-fold; volume was less than 5% of the total volume of cataract surgery at our institution.

## Discussion

With the decrease in volume of refractive surgery over the past few years, some surgeons have had the impression that the clinical characteristics of patients seeking surgery are changing--that patients are older (perhaps because they have more disposable income than younger patients) and that there are more patients with hyperopia or high myopia seeking surgery than before. One rationale is that patients with poor UDVA (e.g., high myopes) are still motivated to have refractive surgery, whereas low myopes (whose UDVA is not as poor) possibly can forgo it. The results of this chart review done at a single academic center over a three-month period in 2007 and in 2009 showed that volume decreased by at least 30% and the distribution of treated refractive errors did not change, but the proportion of high myopes actually decreased when refractive errors were grouped and compared between 2007 and 2009. The proportion of type of procedure--PRK vs. LASIK--has changed, with an increase in the proportion of cases that were PRK between 2007 and 2009. However, there has been no change in mean age of surgical patients.

The estimated overall prevalence rates for refractive errors in the US population 40 years or older in the year 2000 has been described [1]. In short, the estimated prevalence of hyperopia was 9.9%, that of myopia 1 diopter or worse was 25.4%, and that of myopia worse than 5 diopters was 17.4% of all persons with myopia, for a calculated prevalence of 4.5% for myopia worse than 5 diopters (Kempen JH, written communication, September 24, 2010); projected prevalence rates in 2020 are similar. Given these prevalence values, it appears that high myopes are disproportionately represented in refractive surgery. However, the proportion of refractive surgery patients who were high myopes decreased between the summer quarters of 2007 and 2009 at our center. As a group, high myopes would be expected to remain motivated to undergo surgery compared to low myopes even as over all interest in refractive surgery waned. Moreover, patients with high refractive errors (either hyperopia or myopia) may have been dissuaded from surgery before femtosecond laser and wavefront-guided or optimized surgery were available. Assuming a lag between availability of new technology and the time that suitable candidates appear for refractive surgery evaluation, one might expect more patients with high myopia than was observed (or at least expect the proportion to stay the same between 2007 and 2009). However, this did not occur, and the decrease in proportion of high myopia was split between an increase each in the proportions of low myopia and hyperopia, although each increase alone was not statistically significant. The proportion of patients judged not to be good refractive surgery candidates (not including patients with relative contraindication) seems to have dropped from about 50% to 30% in the last decade (unpublished data from our institution), so this change suggests we are offering surgery to candidates who might not have been candidates 5 to 10 years earlier. However, one reason for the decrease in proportion of highly myopic patients might be reaction of surgeons to the FDA advisory panel. Amongst some surgeons, the minimum requirement for residual stromal bed thickness has risen over the years.

One reason we chose the manifest refraction rather than the treated refractive error as a clinical characteristic of interest is that there is an approximate association between UDVA and the magnitude of the spherical equivalent of the manifest refraction. We also believe that preoperative UDVA informs the decision of most patients to have laser refractive surgery, although uncorrected near visual acuity is an additional consideration when a patient contemplates monovision correction. In our series, the fellow eye of patients who chose monovision still underwent surgery to improve UDVA, meaning that UDVA (which can reflect manifest refraction) was still important to patients. In other words, there were no patients who were emmetropic and underwent surgery to attain ametropia--for example, to attain monovision.

Not only was manifest refraction a valid means to stratify patients and also an approximate indication of UDVA, but also in this group of patients, it was similar to the treated refractive error and wavefront refraction. All patients who underwent monovision correction in this series chose "mini" monovision, wherein the goal was 0.75 to 1 diopter of myopia, meaning the difference between manifest and treated spherical equivalents were not dissimilar. Wavefront and manifest refractions are rarely exactly the same, but wavefront refractions are always compared to manifest refractions prior to treatment, and they are not allowed to differ by more than 0.50 to 0.75 diopters, depending on whether the treatment is myopic or hyperopic.

The average proportion of enhancements at our center over the past 10 years has been below 5%; the advent of wavefront technology appears to have contributed. Surprisingly, the proportion increased from 4.8% in the summer quarter of 2007 to 13.3% in the summer quarter of 2009. It is highly unlikely that surgical results became less predictable between 2007 and 2009. Moreover, almost all enhancement cases were internal patients, not ones referred from the community. Although the proportion of hyperopic correction increased between 2007 and 2009 and might have explained the higher enhancement rate in 2009, we found that 50% of the enhancements in 2009 were performed on patients who were previously low myopes, whereas the overwhelming majority of patients undergoing enhancement in 2007 were previously high myopes, The increase in the proportion of cases that were hyperopic between 2007 and 2009 may result in an increased enhancement rate in years following 2009, however, since a lag may occur. Therefore instead of the reason being a higher percentage of hyperopic first-time treatments, this increase in proportion of enhancements is likely a result of the change in over all volume of refractive surgery (the denominator). Surgical volume has decreased worldwide, most likely because of the global financial crisis http://www.crstodayeurope.com/Issues/0309/0309_04.pdf, http://www.aao.org/isrs/resources/trendssurvey.cfm; volume in Europe and the United States has decreased by 20-30% with our center being no exception. Prices of procedures at our center have remained stable. Academic centers have traditionally charged higher prices, most likely due to higher overhead. The average price of our competitors, however, has grown closer to our prices as centers offering far below-market prices have gone out of business. Between 2007 and 2009, our advertising budget shrank as a result of decreased revenue from surgery; the smaller budget may or may not have contributed to further erosion of volume. Moreover, at our center, the surgical fee includes follow-ups and enhancements done within two years of surgery. One possibility is that with the decrease in surgical volume (the denominator), fewer new patients underwent surgery, replaced by a larger percentage of patients who had had previous refractive surgery at our center, who qualified for an enhancement, and for whom cost was no barrier.

We noted a higher proportion of PRK in the summer quarter of 2009 than in 2007. The rise in proportion of PRK cases has been steady since 2000 to 2009, from 3% to 25%. In general, many surgeons are performing more PRK than they did in the past [2]; reasons include concerns about residual stromal bed thickness in LASIK vs. PRK, decreased risk of PRK-associated haze with advances in laser technology, use of mitomycin-C, LASIK flap complications, and findings that PRK induces lower amounts of higher order aberrations than does LASIK, both in conventional [3-5] and wavefront-guided settings [2]. A large review found conventional PRK and LASIK to be comparable in all aspects of visual acuity, although the investigators did not evaluate higher order aberrations [6]. In this series, PRK was performed on high myopes with thin corneas and for primary procedures and enhancements to correct low refractive error. Enhancements were split between PRK and LASIK, such that the higher proportion of enhancements in 2009 cannot account for the increase in PRK.

The proportion of PTK cases decreased drastically. Between 2007 and 2009 at our center, PTK became a self-pay procedure. This arrangement likely had an impact on the number of PTK cases done, although PTK, unlike LASIK and PRK, is used to treat conditions that are not correctable with spectacles or contact lenses. One may therefore argue that PTK should not be compared with purely refractive procedures like LASIK and PRK. However, we chose to examine the distribution of PTK and non-PTK procedures because all are self-pay procedures at our center. Moreover, because PTK is a therapeutic procedure and patients are drawn from the larger community, one would expect the volume of PTK to remain stable. One might even expect the proportion of PTK over the volume of all excimer laser procedures to increase as volume of procedures (denominator) decreased, but instead the proportion of PTK decreased between 2007 and 2009. Very few refractive surgeons in our metropolitan area offer PTK anymore, so it is not clear where patients are seeking treatment if not at our tertiary center and if at all.

Although we expected to see the mean age of patients increase from 2007 to 2009 because older patients might have higher disposable income compared to younger patients, we found there was no difference. More patients in 2009 underwent laser refractive surgery to correct ametropia following cataract extraction with implantation of a presbyopia-correcting lens than did in 2007. A history of cataract extraction would imply an older set of patients, but the increase in the number of post-cataract patients between 2007 and 2009 was outweighed by the relative youth of the other patients undergoing refractive surgery.

Whereas laser refractive surgery is a completely elective procedure, the overwhelming majority of patients undergoing premium (presbyopia- or astigmatism-correcting) intraocular lens (IOL) implantation undergo cataract extraction at the same time; in other words, they are not clear lens exchanges. At Wilmer, when combined with cataract extraction, the out-of-pocket cost (i.e., borne by the patient, not by insurance) of a presbyopia-correcting IOL is the same as LASIK or PRK; the toric IOL is 50% less. Presumably, because these are not clear lens exchanges, these patients might be less price-sensitive. The volume of these IOLs at Wilmer increased manifold between 2007 and 2009. Overall, however, premium IOLs comprised a very small proportion of total cataract volume at Wilmer between 2007 and 2009. Moreover, the volume of these IOLs was at least a magnitude smaller than the volume of laser refractive procedures. Therefore, volume and revenue changes in laser refractive surgery at our center are not being recouped in premium IOLs.

## Conclusions

Several limitations are inherent in this study. A three-month time frame was chosen to attain enough patients to detect trends without obscuration by random fluctuations. One reason the year 2007 was chosen was that it preceded the official start of the economic recession. In addition, many surgeons sense that refractive surgery volume has been decreasing since 2007 (or earlier) and that it continues to decrease. This sentiment persists despite the official end of the recession in December 2009, published by the NBER. Therefore, one critique may be that the time points should be further apart, e.g., 2005 and 2010 or 2009 and 2014, especially since patient/consumer interest may lag changes in the overall economy. Moreover, this three-month survey may be only a snapshot of patient characteristics and procedure types that may or may not be validated by larger studies. In addition, the summer quarter may be different from other quarters for reasons we do not know. Last, patients at an academic center may be quite different from those in the community. Nonetheless this three-month survey done in 2007 and again in 2009 shows that there is a change in the proportion of high myopes, in the types of procedures (PRK vs. LASIK; PTK vs. PRK and LASIK), and in the proportion of enhancement procedures being performed at our center. Premium IOL implantation increased in the same period, but such IOLs still constitute a very small fraction of the total IOLs implanted in our department. A larger study with a longer time frame may be required to show whether there truly has been no change in the distribution of treated refractive errors or in the mean age of patients. Concomitant with such a study might be comparisons with and/or cumulative analysis of data from other academic centers and community practices.

## Competing interests

The author declares that they have no competing interests.

## Authors' contributions

ICK conceived of the design of the study; collected, interpreted, and analyzed the data; and wrote the manuscript, giving final approval of the version to be published

## Pre-publication history

The pre-publication history for this paper can be accessed here:

http://www.biomedcentral.com/1471-2415/11/11/prepub
